# MiR-218 Promotes Adriamycin-Induced H9C2 Apoptosis by Inhibiting Stress-Associated Endoplasmic Reticulum Protein 1

**DOI:** 10.1155/2022/6881103

**Published:** 2022-01-04

**Authors:** Qinghua Chen, Gang Chen, Shuofang Zhao

**Affiliations:** ^1^Department of Cardiology, People's Hospital of Danzhou City, Danzhou, China; ^2^Department of Gynecology and Obstetrics, The Public Health Clinical Center of Chengdu, Chengdu, China; ^3^Guangdong Academy of Medical Sciences and Guangdong Provincial People's Hospital, Guangzhou, China

## Abstract

**Objective:**

Adriamycin is a clinically important chemotherapeutic drug, but its use is restricted due to its myocardial toxicity. Therefore, it is especially important to explore the toxicity mechanism of Adriamycin (ADR) to cardiomyocytes.

**Methods:**

The myocardial toxicity model of ADR was constructed in vitro, and the effect of miR-218 inhibitor and sh-Serp1 on the activity of H9C2 cells induced by ADR was detected by MTT method. Also, flow cytometry, real-time polymerase chain reaction (RT-PCR), and TUNEL staining were used to detect the cell apoptosis. The activity of LDH was detected by colorimetry, and the interaction of miR-218 with Serp1 was detected by double-luciferase reporter gene assay. Western blotting technique was used to detect the expression level of caspase3 and p38 MAPK signal pathway.

**Results:**

miR-218 inhibitor can obviously inhibit ADR-induced decrease in cell activity of H9C2 cells, inhibit cell apoptosis, and inhibit p38 MAPK signaling pathway activation. Conversely, sh-Serp1 aggravated the decrease in H9C2 cell activity and promoted cell apoptosis.

**Conclusion:**

Upregulation of miR-218 expression will promote ADR-induced apoptosis of H9C2 cells. At the same time, we confirmed that the mechanism by which miR-218 promotes myocardial apoptosis was through the Serp1/p38 MAPK/caspase-3 signaling pathway.

## 1. Introduction

The discovery of malignant tumor chemotherapy drugs—“anthracycline antibiotics”—is a major success in the history of cancer treatment, especially for the treatment of pediatric tumors. More than 50% of the survivors who had suffered from cancer in childhood had been treated with onion ring antibiotics. And with the use of this type of chemotherapy drugs, the 5-year survival rate of pediatric tumors has increased from about 30% in the sixties to the current 70%-80% [1, 2]. However, the clinical use of ADR is limited by the cardiac toxic side effects it can cause, mainly manifested as congestive cardiomyopathy [3]. At the end of the 1970s, the first retrospective clinical study confirmed that cardiac dysfunction can be directly attributed to the repeated accumulation of ADR. The changes in heart function caused by ADR are similar to dilated cardiomyopathy, and once the disease progresses, the prognosis is usually very poor, often lethal, and the current treatment methods seem to improve the prognosis. Although there is already a lot of evidence to give hypotheses about the mechanism of ADR's cardiac toxic side effects from different aspects, people still do not fully understand its precise cause. The cell death mechanism is considered to be an important mechanism for ADR-induced cardiomyopathy. Many in vivo and external experiments over the past decades have shown that the cardiac toxicity caused by ADR is related to the apoptosis or necrosis of myocardial cells [4, 5].

In recent years, miRNA has been a hot spot in medical research. miRNA is a single-stranded noncoding small RNA composed of 21-25 nucleotides, which mainly mediates the posttranscriptional degradation of target genes or inhibits their translation, thereby affecting a variety of pathophysiological processes including development, differentiation, proliferation, and apoptosis in the course of life [6, 7]. miRNAs are widely distributed in the genomes of animals and plants, and their expression is highly conservative, time-series, and tissue-specific. It can regulate about 30% of human protein-related genes. Therefore, changes in miRNA expression levels directly participate in important pathophysiological processes that regulate the occurrence and development of many cardiovascular diseases (CVD), such as myocardial hypertrophy, angiogenesis, arrhythmia, myocardial ischemia, myocardial apoptosis, and myocardial fibrosis [8, 9]. At present, multiple miRNAs have been confirmed to be involved in the process of myocardial apoptosis. Some studies have found that miR-1 expression is increased in ischemic cardiomyocytes and plays a key role in apoptosis. Its expression level is proportional to the degree of heart injury [10]. Another study showed that miR-1 and miR-133 can regulate oxidative stress-induced apoptosis in rat cardiomyocytes, miR-1 promotes apoptosis, and miR-133 inhibits apoptosis [11]. However, we still do not know the specific role of miRNA regulation in cardiovascular disease. We need to further study the disease-associated miRNAs and the target genes, so as to clarify the effects of miRNAs on CVD. Whether these miRNAs are upregulated or downregulated, they may become new molecular targets for the treatment of CVD.

Serp1, as one of the important chaperone proteins of unfolded protein response that can alleviate endoplasmic reticulum stress damage, has been shown to play a crucial role in alleviating tissue apoptosis caused by endoplasmic reticulum stress [12]. However, the role and mechanism of Serp1 in CVD are still unclear. Therefore, in this study, we will explore its role in ADR-induced apoptosis of cardiomyocytes.

MAPKs are key apoptosis regulating proteins that can transduce various extracellular signals into intracellular pathophysiological processes. There are three main enzymes in the MAPK family, including extracellular signal-regulated kinase (ERK), c-Jun NH2-terminal kinase, and p38 MAPK. p38 MAPK, a serine/threonine enzyme, can be activated by various environmental stimuli. A large number of studies have confirmed that p38 MAPK activation may be related to cardiomyocyte apoptosis [13]. Therefore, inhibiting the activation of p38 MAPK has a good therapeutic effect on cardiovascular disease and can reduce myocardial cell necrosis and apoptosis, inhibit downstream caspase-3 activation, and improve heart function. Caspases are an important member of the aspartate-specific cysteine protease family and are widely expressed in various mammalian cells. Caspase-3, as a basic agonist of apoptosis and a crucial effector, plays a key role in performing the intrinsic apoptotic protease cascade and the external apoptotic pathway [14]. This indicates that p38 MAPK plays a crucial role in cardiomyocyte apoptosis. Therefore, we speculate that p38 MAPKs also have a similar regulatory effect on ADR-induced cardiomyocyte apoptosis.

## 2. Materials and Methods

### 2.1. Cell Culture

Rat H9C2 cells were purchased from the American type culture collection (ATCC, Manassas, VA, USA). The cell culture medium was Dulbecco's modified eagle medium (DMEM, Life Technology, Wuhan, China) containing 10% fetal bovine serum (FBS, Life Technology, Wuhan, China) and 1% penicillin (Life Technology, Wuhan, China). The cells will be passaged 1-2 times before transfection to ensure the cells grow vigorously. Approximately 12 hours before transfection, cells were passaged from culture flasks at 80% density and plated into culture dishes (Life Technology, Wuhan, China).

### 2.2. Transfection and Cell Processing

The design and synthesis of miR-218 inhibitor, inhibitor NC, shRNA Serp1, and shRNA-NC were completed by Shanghai Gene Pharma. H9C2 cells were plated in 12-well plates at a density of 3‐5 × 10^5^/mL and transfected after 48 hours of culture. Before transfection, the cell culture medium was replaced with 900 *μ*L of complete DMEM medium (without antibiotics). Next, we transfected cells according to the Lipofectamine^2000^ instructions (Gene Pharma, Shanghai, China). After the transfection, the cells were transferred to a 37°C, 5% CO_2_ incubator for 8 hours, and then, the culture medium was replaced with fresh complete DMEM culture medium and placed in the incubator for 48 hours. According to literature reports, we selected 1 *μ*M ADR treatment model group. The control group used phosphate-buffered saline (PBS) for treatment.

### 2.3. MTT (3-(4,5-Dimethylthiazol-2-Yl)-2,5-Diphenyl Tetrazolium Bromide) Experiment

The cultured H9C2 cells were seeded in 96-well culture plates, and after transfection and 1 *μ*M ADR treatment, MTT solution (R&D Systems, Minneapolis, MN, USA) was added to each well. The cells continued to incubate for 4 hours in the incubator, then the supernatant of the 96-well plate was carefully discarded. 150 *μ*L dimethyl sulfoxide (DMSO) (R&D Systems, Minneapolis, MN, USA) was added to each well and shaked for 10 minutes to fully melt the crystals. The activity of myocardial cells was detected, and the absorbance value of each well was measured by enzyme-linked immunoassay (Thermo Fisher Scientific, Waltham, MA, USA).

### 2.4. Lactate Dehydrogenase (LDH) Activity

After centrifugation, the supernatant was transferred to the new EP tube. 25 *μ*L matrix buffer and 5 *μ*L coenzyme I solution were added to the EP tube. After mixing, the supernatant was bathed at 37°C for 15 minutes, then 25 *μ*L 2, 4-dinitrobenzene hydrazine was added, 37°C water bath for 15 minutes after blending. 25 *μ*L, 0.4 mol/L sodium hydroxide solution was blended and placed 5 minutes at room temperature and 450 nm wavelength determination of optical density (OD) value. The LDH activity was calculated according to the formula (Jian Cheng, Nanjing, China).

### 2.5. Western Blotting (WB) Technology

H9C2 cells were lysed by radioimmunoprecipitation assay (RIPA) lysate (Camilo Biological, Nanjing, China) containing phenylmethylsulfonyl fluoride (PMSF) to extract proteins. 30 *μ*g protein in each group was separated by 10% sodium dodecyl sulphate-polyacrylamide gel electrophoresis (SDS-PAGE). Then, a polyvinylidene difluoride (PVDF; Thermo Fisher Scientific, Waltham, MA, USA) membrane was used to transfer the separated protein, then, blocked with 5% skim milk, incubated the primary antibody (Caspase3, Abcam, Cambridge, MA, USA, Rabbit, 1 : 1000; Serp1, Abcam, Cambridge, MA, USA, Mouse, 1 : 2000; p38, Abcam, Cambridge, MA, USA, Rabbit, 1 : 1000; P-p38, Abcam, Cambridge, MA, USA, Mouse, 1 : 2000; GAPDH, Abcam, Cambridge, MA, USA, Mouse, 1 : 5000) at 4°C overnight; the next day, added with secondary antibodies (goat anti-rabbit IgG antibody, Yifei Xue Biotechnology, Nanjing, China, 1 : 2000) and incubated at room temperature for 1 hour. The enhanced chemiluminescence (ECL) technology (Thermo Fisher Scientific, Waltham, MA, USA) was used to develop immunoblots, and GAPDH was used as an internal reference.

### 2.6. RNA Isolation and Real-Time Polymerase Chain Reaction (RT-PCR)

After corresponding treatment, the H9C2 cells were washed twice with phosphate-buffered saline (PBS), 500 *μ*L TRIzol reagent (Thermo Fisher Scientific, Waltham, MA, USA) was added to each well of the 12-well plate, and total RNA was extracted according to the kit instructions, measured the RNA concentration by ultraviolet absorption method, and read the absorbance at 260 nm and 280 nm of the spectrophotometer. If the A260/280 ratio of the RNA solution was in the range of 1.6-2.0, the extracted RNA can be reverse transcribed. The reaction conditions were as follows: 25°C, 10 minutes, 37°C, 120 minutes, 85°C, 5 minutes. Next, we used the steps of Applied Biosystems' SYBR Green PCR Master Mix kit (R&D Systems, Minneapolis, MN, USA) for polymerase chain reaction. GAPDH was considered as an internal reference for mRNA. The analysis of experimental results uses the 2^−ΔΔCt^ method. Primer sequence is shown in Table 1.

### 2.7. TUNEL Staining

Place the sterile slides in a 24-well plate, and H9C2 cells were seeded onto the coverslip of the 24-well plate according to the culture method described above and placed in a cell culture box for cultivation. After the H9C2 cells received the corresponding treatment, the cell slides were washed three times with prechilled PBS for 5 minutes each time. 200 *μ*L of 4% paraformaldehyde (Jian Cheng, Nanjing, China) was added to each well to fix the cardiomyocytes on the slide and fixed at room temperature for 10 minutes. Then, the H9C2 cells were washed twice with PBS for 5 minutes each time and treated the slides with Proteinase K working solution (R&D Systems, Minneapolis, MN, USA) for another 20 minutes. After washing the H9C2 cells twice with PBS, the TUNEL reaction mixture (R&D Systems, Minneapolis, MN, USA) was added dropwise to the slide and incubated for 1 hour at room temperature. Then, it was washed twice with PBS, and the nuclei were stained with DAPI reagent (Elabscience, Wuhan, China) in the dark room for 10 minutes, finally, observed under a fluorescence microscope (R&D Systems, Minneapolis, MN, USA).

### 2.8. Double-Luciferase Reporter Gene Assay

Luciferase plasmids (R&D Systems, Minneapolis, MN, USA) containing wild-type and mutant Serp1 mRNA 3′UTR were cotransfected with miR-218 mimics and miR-218 NC into 293 T cells (ATCC, Manassas, VA, USA), respectively. After transfection for 48 hours, 293 T cells were lysed, and the firefly luciferase activity and Renilla luciferase activity were detected, respectively.

### 2.9. Annexin V-FITC/PI Double-Staining Flow Cytometry to Detect Apoptosis Rate

After the H9C2 cells were treated according to the experimental design, the cells were digested with trypsin (Sinopharm Chemical Reagent, Shanghai, China) without EDTA (ethylenediaminetetraacetic acid). After centrifugation, the cells were washed twice with prechilled PBS. After centrifugation again, the supernatant was discarded, and 500 *μ*L of 1× binding buffer (Sinopharm Chemical Reagent, Shanghai, China) was added to resuspend the cells. After adding 5 *μ*L fluorescein 5-isothiocyanate (FITC) and mixed well, such was incubated at room temperature in the dark for 15 minutes. Then, 3 *μ*L propidium iodide (PI) was added to mix and incubate for 5 minutes in the dark. Finally, the cells were transferred to a sample tube for instrumental analysis.

### 2.10. Statistical Analysis

GraphPad Prism21.0 (La Jolla, CA, USA) was used to perform statistical analysis on the data. The experimental data was expressed as mean ± standard deviation (mean ± SD). The comparison of the two groups of data was performed by independent sample *t*-test. Analysis of variance was used for comparison among multiple groups. Take *p* < 0.05 as the standard of significant difference.

## 3. Results

### 3.1. ADR Induces Apoptosis of H9C2 Cells

**Figure 1:**
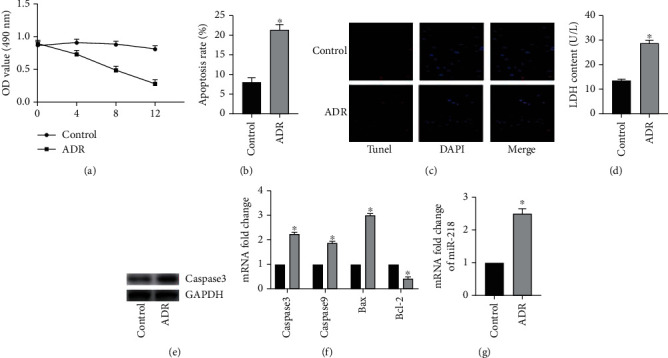
ADR induces apoptosis of H9C2 cells. (a) The cell activity was detected by MTT method, *n* = 6. (b) The apoptosis level of cell was detected by flow cytometry, *n* = 3. (c) TUNEL staining, *n* = 3. (d) The activity of LDH detected by the colorimetry method, *n* = 3. (e) The protein expression of caspase3 was detected by Western blot, *n* = 3. (f) The mRNA level of caspase3, caspase9, Bax, and Bcl-2 was detected by RT-PCR, *n* = 3. (g) The mRNA level of miR-218 was detected by RT-PCR, *n* = 3 (“^∗^” versus the control group, *p* < 0.05).

According to previous reports in the literature, excessive ADR in the human body activates exogenous and endogenous apoptosis pathways, thereby inducing cardiomyocyte apoptosis. In order to test the success of the in vitro model, we first measured the cell activity of two groups by MTT (Figure 1(a)). As a result, it was found that after treatment of H9C2 cells with 1 *μ*M ADR, the cell activity decreased obviously with the incubation time. Immediately, we used Annexin V for FITC labeling and flow cytometry to detect the apoptosis rate (Figure 1(b)). The test results found that compared with the control group, the percentage of Annexin V-FITC-positive cells in H9C2 cells after 12 hours of ADR treatment increased obviously. At the same time, TUNEL staining also obtained similar results (Figure 1(c)). Subsequently, we found that after ADR treatment of H9C2 cells, the LDH content in the cell supernatant was obviously increased, confirming that ADR treatment of H9C2 cells would induce increased cytotoxicity (Figure 1(d)). Next, we examined the key molecule caspase3 in the apoptosis pathway and found that the expression of caspase3 protein in the ADR group was obviously increased (Figure 1(e)). At the same time, RT-PCR results also found that the ADR group caspase3, caspase9, and Bax also increased obviously at the transcription level, while the expression of antiapoptotic molecule Bcl-2 was obviously reduced (Figure 1(f)). In summary, our preliminary results are consistent with previous studies. In addition, we also found that miR-218 expression was obviously increased in the ADR group, suggesting that miR-218 may be involved in the process of apoptosis (Figure 1(g)).

### 3.2. Knocking down miR-218 Expression Can Alleviate ADR-Induced Apoptosis in H9C2 Cells

**Figure 2:**
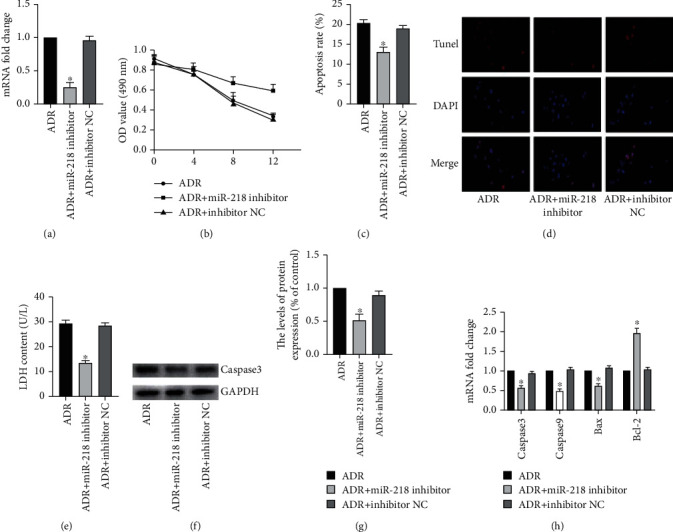
Knocking down miR-218 expression can alleviate ADR-induced apoptosis in H9C2 cells. (a) The mRNA level of miR-218 was detected by RT-PCR. (b) The cell activity was detected by MTT method, *n* = 6. (c) The apoptosis level of cell was detected by flow cytometry, *n* = 3. (d) TUNEL staining, *n* = 3. (e) the activity of LDH detected by the colorimetry method, *n* = 3. (f) The protein expression of caspase3 was detected by Western blot. (g) Gray value analysis of protein strips, *n* = 3. (h). The mRNA level of caspase3, caspase9, Bax, and Bcl-2 was detected by RT-PCR, *n* = 3 (“^∗^” versus the ADR group, *p* < 0.05).

In order to examine the effect of miR-218 on apoptosis of H9C2 cells, based on the above results, we first transfected H9C2 cells with miR-218 inhibitor to downregulate the expression of endogenous miR-218 and verified the transfection efficiency by RT-PCR (Figure 2(a)). Then, MTT detected the cell activity of each group and found that the cell activity of the ADR + miR-218 inhibitor group was higher than that of the ADR and ADR + inhibitor NC groups (Figure 2(b)). At the same time, the results of flow cytometry and TUNEL staining found that the apoptosis rate of the ADR + miR-218 inhibitor group was obviously lower than that of the other two groups (Figures 2(c) and 2(d)). Next, we used colorimetry to detect cytotoxicity. The results showed that the cytotoxicity of the ADR + miR-218 inhibitor group was obviously reduced (Figure 2(e)). Then, we used WB and RT-PCR to detect apoptosis-related molecules to observe the effect of miR-218 on cell apoptosis (Figures 2(f)–2(h)). The results showed that compared with the ADR and ADR + inhibitor NC groups, the expression of caspase3 protein in the ADR + miR-218 inhibitor group was reduced. At the same time, the transcription levels of caspase3, caspase9, and Bax were also obviously reduced, while the expression of antiapoptotic molecule Bcl-2 was obviously increased.

### 3.3. Interaction of miR-218 and Serp1

**Figure 3:**
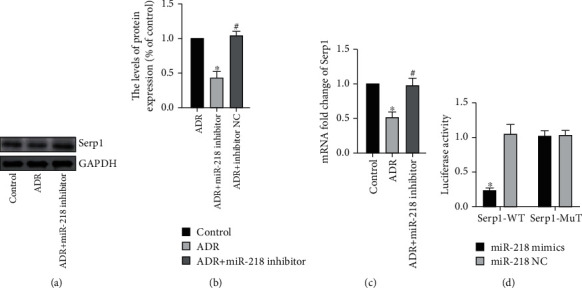
Interaction of miR-218 and Serp1. (a) The protein expression of Serp1 was detected by Western blot. (b) Gray value analysis of protein strips, *n* = 3. (c) The mRNA level of Serp1 was detected by RT-PCR, *n* = 3. (d) luciferase gene report experiment, *n* = 3 (“^∗^” versus the control group, *p* < 0.05; “^#^” versus the ADR group, *p* < 0.05).

First, we predicted through the targetscan website that miR-218 and Serp1 have binding sites. Next, we detected by WB and RT-PCR that Serp1 expression in the ADR group was obviously reduced, while in the ADR + miR-218 inhibitor group, the Serp1 expression was higher than the ADR group (Figures 3(a)–3(c)). At the same time, the results of the luciferase gene report experiment also confirmed our speculation that the fluorescence activity was the lowest in the miR-218 mimics + Serp1-WT group (Figure 3(d)).

### 3.4. Inhibiting Serp1 Promotes ADR-Induced Apoptosis of H9C2 Cells

**Figure 4:**
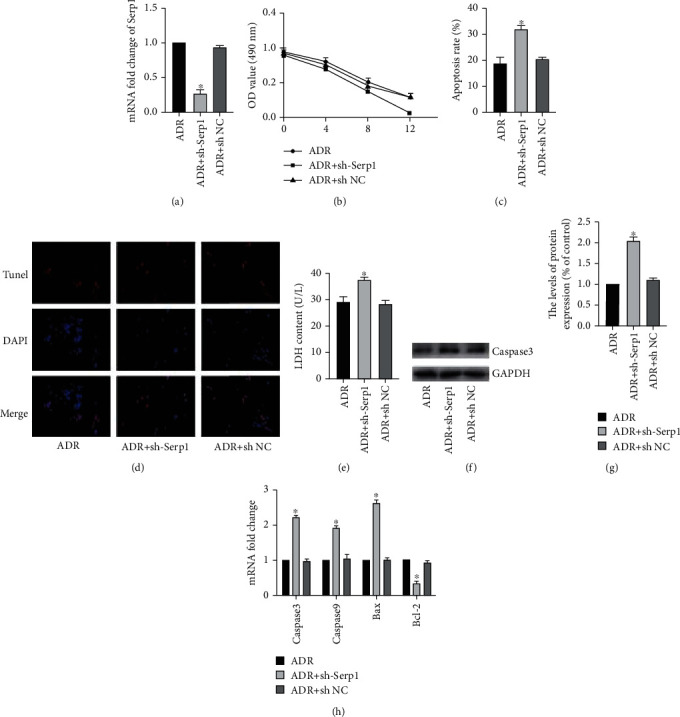
Inhibiting Serp1 promotes ADR-induced apoptosis of H9C2 cells. (a) The mRNA level of Serp1 was detected by RT-PCR, *n* = 3. (b) The cell activity was detected by MTT method, *n* = 6. (c) The apoptosis level of cell was detected by flow cytometry, *n* = 3. (d) TUNEL staining, *n* = 3. (e) The activity of LDH detected by the colorimetry method, *n* = 3. (f) The protein expression of caspase3 was detected by Western blot. (g) Gray value analysis of protein strips, *n* = 3. (h) The mRNA level of caspase3, caspase9, Bax, and Bcl-2 was detected by RT-PCR, *n* = 3 (“^∗^” versus the ADR group, *p* < 0.05).

Based on the above results, we first transfected H9C2 cells with sh-Serp1 and sh-NC and then incubated H9C2 cells with 1 *μ*M ADR for 12 hours. And the transfection efficiency was detected by RT-PCR (Figure 4(a)). Next, MTT found that interfering with Serp1 expression accelerated ADR-induced decrease in H9C2 cell activity (Figure 4(b)). At the same time, flow cytometry and TUNEL staining to detect the apoptosis rate also found that interference with Serp1 expression increased the ADR-induced apoptosis of H9C2 cells (Figures 4(c) and 4(d)). In addition, the cytotoxicity of the ADR + sh Serp1 group also increased obviously (Figure 4(e)). Next, in order to detect the effect of interference with Serp1 on apoptosis, we detected apoptosis-related molecules by WB and RT-PCR (Figures 4(f)–4(h)). The results showed that the caspase3 protein in the ADR + sh Serp1 group was obviously increased. At the same time, the transcription levels of caspase3, caspase9, and Bax were also obviously increased, while the expression of Bcl-2 was obviously reduced.

### 3.5. Effect of miR-218 on p38 MAPK Pathway

**Figure 5:**
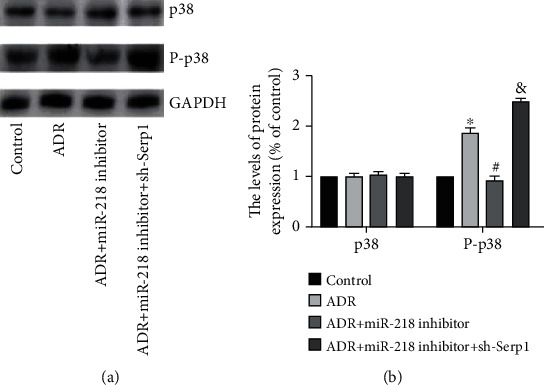
Effect of miR-218 on p38 MAPK pathway. (a) The protein expression of p38 and P-p38 was detected by Western blot. (b) Gray value analysis of protein strips, *n* = 3 (“^∗^” versus the control group, *p* < 0.05; “^#^” versus the ADR group, *p* < 0.05; “^&^” versus the ADR+miR-218 inhibitor group, *p* < 0.05).

The activation of p38 MAPK will lead to cardiomyocyte apoptosis. Therefore, we detected the expression of p-38 and P-p38 protein in H9C2 cells of each group (Figures 5(a) and 5(b)). WB results showed that there was no significant difference in the total p38 of each group. Compared with the control group, the P-p38 protein expression in the ADR group was obviously increased. When we inhibited miR-218 expression, the ADR-induced P-p38 protein expression was obviously reduced, and after interfering with Serp1 expression, P-p38 protein expression was higher than that in the ADR + miR-218 inhibitor group. In summary, the mechanism by which miR-218 promotes H9C2 cells apoptosis was targeted inhibition of Serp1, thereby activating p38 MAPK signaling pathway.

## 4. Discussion

ADR is an antitumor chemotherapy drug. However, due to its toxic effect on the heart, it has been clinically restricted. At present, the mechanism of ADR's cardiotoxicity is roughly divided into (1) ion and free radical hypothesis. The hypothesis believes that the enhancement of oxidative stress and the lack of antioxidants may play a crucial role in the occurrence of cardiomyopathy and heart failure; (2) metabolic mechanism; (3) apoptosis mechanism. Many CVD including ADR-induced cardiomyopathy have reported apoptosis and reduction of cardiomyocytes [3, 15]. Moreover, the above-mentioned intracellular changes will lead to apoptosis of cardiomyocytes, and apoptosis is currently considered to be one of the important mechanisms of ADR-induced cardiomyopathy. Cardiomyocytes are terminally differentiated cells, and the reduction of cardiomyocytes caused by cell necrosis or apoptosis caused by antitumor drugs may cause insufficient cardiac contractility. This may be another reason why the heart is more sensitive to ADR than other tissues [16]. In addition, apoptosis is a highly conservative, strictly regulated, energy-dependent active cell death process, which is essential for normal tissue development and homeostasis [17]. Typical morphological changes of apoptosis include cell and chromatin condensation, DNA fragmentation, and formation of apoptotic bodies. At present, it is believed that there are two classic pathways, including exogenous and endogenous pathways to mediate the transmission of apoptosis signals. Both of these pathways will eventually activate the downstream executor caspases 3. Under different cell types and death stimulation signals, the precise molecular regulation mechanism of caspases will be different. Therefore, the apoptosis of cardiomyocytes caused by ADR is also different from that of tumor cells [18].

miRNA is a type of small noncoding RNA that can mediate gene silencing at the posttranscriptional level. Therefore, miRNA plays a key regulatory role in biological processes such as cell proliferation, differentiation, apoptosis, and stress response [19]. For example, miR-19b and miR-499 inhibited hydrogen peroxide-induced cardiomyocyte apoptosis through their respective target genes [20]. MiR-24 can inhibit the apoptosis of myocardial cells during myocardial infarction and reduce the infarct size [21]. In this study, it was found that miR-218 is upregulated in myocardial cells incubated with ADR, and ADR can cause obvious apoptosis of H9C2 cells. We proposed the hypothesis that miR-218 participates in the regulation of cardiomyocyte apoptosis. To further explore this issue, we used transfected miR-218 inhibitor to achieve downregulation of miR-218 in H9C2 cells. After 12 hours of ADR treatment, flow cytometry and TUNEL staining were used to detect cardiomyocyte apoptosis. Both results found that after miR-218 expression was downregulated, H9C2 cells showed less apoptosis after ADR treatment. Next, we predicted through the tagscan website that Serp1 and miR-218 have binding sites, and Serp1 has a role in regulating apoptosis. From this, we speculate that miR-218 may regulate apoptosis by regulating Serp1. Therefore, after transfecting sh-Serp1, we continue to explore its mechanism of action. The results showed that when we interfered with Serp1 expression, it increased the H9C2 cell apoptosis induced by ADR. Combined with the above results, we demonstrated that miR-218 negatively regulates Serp1, thereby promoting apoptosis of H9C2 cells.

A large number of studies have proved that the activation of p38 MAPK phosphorylation plays a crucial role in ischemia-induced cardiomyocyte apoptosis. Recent studies have found that miR-146a directly inhibits the activation of p38MAPK by directly targeting TRAF6, thereby inhibiting cardiomyocyte apoptosis induced by ischemia and hypoxia [22]. Whether miR-218 promotes ADR-induced cardiomyocyte apoptosis via the p38 MAPK pathway has not been reported. Therefore, we conducted research on this. Our results found that we found that after treatment of H9C2 cells with ADR, miR-218 expression increased, and Serp1 expression decreased, thereby promoting the increase in apoptosis rate. When we inhibited miR-218 expression, the apoptosis rate decreased, and the p38 MAPK pathway was suppressed. Conversely, when we interfere with Serp1 expression, the apoptosis rate increases, and the p38 MAPK pathway was activated.

## 5. Conclusion

This study clarifies that upregulation of miR-218 expression will promote ADR-induced cardiomyocyte apoptosis. Furthermore, we confirmed that the mechanism by which miR-218 promotes myocardial apoptosis is through the Serp1/p38 MAPK/caspase-3 signaling pathway. Therefore, our research will provide a new treatment strategy for the treatment of ADR toxic cardiomyopathy.

## Figures and Tables

**Table 1:** RT-PCR primers.

Gene name	Forward (5′ > 3′)	Reverse (5′ > 3′)
MiR-218	AGGGGTTGTGCTTGATCTAA	GTTGTGGTTGGTTGGTTTGT
Serp1	CCCTGGTTATTGGCTCTCT	ACTTCACATGCCCATCCT
Bax	GAGGTCTTCTTCCGTGTGG	GATCAGCTCGGGCACTTT
Bcl-2	GTCGCTACCGTCGTGACTTC	CAGACATGCACCTACCCAGC
Caspase3	ATGGAGAACAACAAAACCTCAGT	TTGCTCCCATGTATGGTCTTTAC
Caspase9	TCCTGGTACATCGAGACCTTG	AAGTCCCTTTCGCAGAAACAG
GAPDH	ACAACTTTGGTATCGTGGAAGG	GCCATCACGCCACAGTTTC

RT-PCR, quantitative reverse-transcription polymerase chain reaction.

## Data Availability

The datasets used and analyzed during the current study are available from the corresponding author on reasonable request.
